# Nanoemulsion-Enabled Oral Delivery of Novel Anticancer ω-3 Fatty Acid Derivatives

**DOI:** 10.3390/nano8100825

**Published:** 2018-10-13

**Authors:** Gabriela Garrastazu Pereira, Tristan Rawling, Michele Pozzoli, Curtis Pazderka, Yongjuan Chen, Colin R. Dunstan, Michael Murray, Fabio Sonvico

**Affiliations:** 1 National Council for Scientific and Technological Development—CNPq, Brasília 71605-001, Brazil; garrastazugroup@gmail.com; 2 Discipline of Pharmacy, Graduate School of Health, University of Technology Sydney, Ultimo, NSW 2007, Australia; michele.pozzoli@student.uts.edu.au; 3 School of Mathematical and Physical Sciences, Faculty of Science, University of Technology Sydney, Ultimo, NSW 2007, Australia; tristan.rawling@uts.edu.au (T.R.); curtis.pazderka@uts.edu.au (C.P.); 4 School of Aerospace, Mechanical and Mechatronic Engineering, University of Sydney, Sydney, NSW 2006, Australia; yong.chen@sydney.edu.au (Y.C.); colin.dunstan@sydney.edu.au (C.R.D.); 5 Discipline of Pharmacology, School of Medical Sciences, Sydney Medical School, University of Sydney, Sydney, NSW 2006, Australia; michael.murray@sydney.edu.au

**Keywords:** nanoemulsion, oral delivery, ω-3 polyunsaturated fatty acid derivative, MDA-MB-231, triple-negative breast cancer

## Abstract

Lipid-based drugs are emerging as an interesting class of novel anticancer drugs with the potential to target specific cancer cell metabolic pathways linked to their proliferation and invasiveness. In particular, ω-3 polyunsaturated fatty acids (PUFA) derivatives such as epoxides and their bioisosteres have demonstrated the potential to suppress growth and promote apoptosis in triple-negative human breast cancer cells MDA-MB-231. In this study, 16-(4′-chloro-3′-trifluorophenyl)carbamoylamino]hexadecanoic acid (ClFPh-CHA), an anticancer lipid derived from ω-3,17,18-epoxyeicosanoic acid, was formulated as a stable nanoemulsion with size around 150 nm and narrow droplet size distribution (PDI < 0.200) through phase-inversion emulsification process followed by high pressure homogenization in view of an oral administration. The ClFPh-CHA-loaded nanoemulsions were able to significantly decrease the relative tumor volume in mice bearing an intramammary tumor xenograft at all doses tested (2.5, 10 and 40 mg/kg) after 32 days of daily oral administration. Furthermore, absolute tumor weight was decreased to 50% of untreated control at 10 and 40 mg/kg, while intraperitoneal administration could achieve a significant reduction only at the highest dose of 40 mg/kg. Results suggest that oral administration of ClFPh-CHA formulated as a nanoemulsion has a sufficient bioavailability to provide an anticancer effect in mice and that the activity is at least equal if not superior to that obtained by a conventional parenteral administration of equivalent doses of the same drug.

## 1. Introduction

Breast cancer is the most common type of cancer among women worldwide after non-melanoma skin cancer, affecting more than 200,000 women annually and killing more than 40,000 women each year. Breast cancer also affects men, but it is rare, accounting for only 1% of all cases of cancer. Quite worryingly, statistics indicate an increasing incidence in both developed and developing countries. There are several types of breast cancer, and their characteristics affect both treatment options and therapeutic outcomes. Triple-negative breast cancer (TBNC), i.e., tumors that lack expression of estrogen receptor (ER), progesterone receptor (PR), as well as human epidermal growth factor receptor type 2 (HER2), are aggressive and highly metastatic, and do not respond to endocrine or monoclonal antibodies-based therapies. Thus, patients with TNBC have limited treatment options, and subsequently, a poorer prognosis [1]. It is known that during the development of breast cancer several regulatory mechanisms are in imbalance, while processes such as chronic inflammation and pro-oxidative reactions are stimulated. The imbalance between free radicals and antioxidant species induced by exogenous (diet and smoking) or endogenous (estrogens) factors promotes oxidative stress during the onset, promotion and progression of breast cancer [2,3,4]. The development of compounds with novel mechanisms of action against tumor cells are required to develop anticancer therapeutics able to induce further remissions in cancer patients who are resistant to established agents.

**Figure 1:**
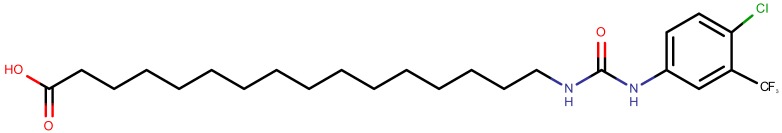
Structure of the ω-3 polyunsaturated fatty acid derivative 16-(4′-chloro-3′-trifluorophenyl)carbamoylamino]hexadecanoic acid (ClFPh-CHA).

Many studies have recently evidenced a relationship between polyunsaturated fatty acids and cancer. In fact, ω-6 polyunsaturated fatty acids (PUFAs) are converted by tumor cells to potent eicosanoid promoters of tumor cell proliferation through the over-expression of cyclooxygenase, lipoxygenase or cytochrome P450 enzymes. In contrast, several eicosanoid metabolites from the biotransformation of ω-3 PUFAs impair particular tumorigenic pathways, indicating that the intake of ω-3 polyunsaturated fatty acids might decrease cancer risk, in particular for breast, prostate, and colon cancer [5,6]. These anticancer effects, attributed to ω-3 PUFAs metabolites such as prostaglandin E3, resolvins, 15-hydroxyeicosatetraenoic acids (HETE), epoxides of docosahexaenoic (DHA), and 17,18-epoxide of eicosapentaenoic acid (ω-3,17,18-epoxyeicosanoic acid) have been the trigger to develop synthetic ω-3 PUFA epoxides derivatives and to study their anti-proliferative and pro-apoptotic effect in breast cancer models [7,8,9,10]. From this work, the synthetic ω-3 polyunsaturated fatty acid derivative 16-(4′-chloro-3′-trifluorophenyl)carbamoylamino]hexadecanoic acid (ClFPh-CHA, Figure 1) was identified as a promising anticancer agent [10].

However, one of the main problems with this class of compounds is their solubilization, as most of them show very poor water solubility making their administration challenging. Formulation is a promising approach to overcome the active compound physico-chemical issues to improve bioavailability and enable their clinical use. In this direction, an interesting approach emerging as a possible solution for the administration of such challenging compounds is the use of nanoemulsions [11,12].

Nanoemulsions (NEs) are colloidal dispersions, which consist in emulsions having the dispersed droplets in the nanometric scale. Droplet size falls typically between those of microemulsions and conventional emulsions, with a size range of 20–500 nm. In contrast to microemulsions that are thermodynamically stable, nanoemulsions are subjected the same thermodynamic instability of macroemulsions. However, nanoemulsions are more stable than conventional emulsions, since their small droplet size makes them less susceptible to creaming or sedimentation phenomena and provides them with high kinetic stability. Another important aspect is that they can be produced using more practical surfactant concentrations, e.g., 5%, when compared to ~50%, which is typically used to prepare microemulsions [13]. Accordingly, submicron and nanoemulsions have gained increasing attention as drug delivery systems for oral, parenteral, transdermal, and topical (e.g., dermo-cosmetic, vitamins, and anti-aging agents) applications [14,15,16,17]. Recent progress in the control of size distribution and in the understanding of stabilization mechanisms of nanoemulsion has also contributed to renewed attention to these particular emulsion systems [18,19]. 

In particular, in the case of oral administration nanoemulsions, they have displayed some specific advantages: (1) NEs, because of drug encapsulation, provide a platform to protect active pharmaceutical substances from enzymes, low pH and other environmental conditions in the gastrointestinal (GI) tract; (2) oral delivery via nanoemulsion results in rapid absorption and steady state levels are reached within 30 min, which suggests substantial absorption even from P-glycoprotein-rich distal ileal regions; (3) NEs have been shown to be able to increase the oral bioavailability of lipophilic drugs (such as curcumin, some antibiotics, fatty acids, etc.); and (4) NEs can also minimize side effects with a reduction of dose with the same effect as a non-encapsulated drug.

The aim of the present work was to design, develop, and characterize a nanoemulsion loaded with the novel synthetic fatty acid ClFPh-CHA that induces apoptosis in TNBC cell lines. The loaded nanoemulsion was then used for oral delivery in an animal tumor model, and its anticancer activity was compared to that of the same compound administered intraperitoneally.

## 2. Materials and Methods

### 2.1. Materials

Sorbitan monooleate (Span™ 80) (HLB = 4.3) and Polysorbate 80 (Tween™ 80) (HLB = 15.0) were both supplied by Croda (Wetherill Park, NSW, Australia). The oil phase in the emulsion was constituted by pharmaceutical grade medium chain triglycerides (Labrafac™ lipophile WL 1349, Gatefossè, Saint-Priest, France). Ultrapure Milli-Q water filtered through 2 µm filters was used in all experiments (Arium^®^ pro—Water purification system, Sartorius, Dandenong South, VIC, Australia). The ω-3 17,18-epoxyeicosanoic acid analogue [16-(4′-chloro-3′-trifluorophenyl)carbamoylamino]hexadecanoic acid (ClFPh-CHA, Figure 1) was synthesized according to a procedure previously reported by Rawling et al. [10]. HPLC grade acetonitrile and methanol for chromatography were obtained from Honeywell Burdick and Jackson (Muskegon, MI, USA). Ammonium formate AR grade was obtained from Asia Pacific Specialty Chems Ltd. (Seven Hills, NSW, Australia). Chemicals were used as received without any further purification.

### 2.2. Methods

#### 2.2.1. Study of the Impact of Process Parameters on Nanoemulsion Preparation

To determine the influence of different process and composition variables on the nanoemulsion preparation, several conditions parameters were investigated.

(a) Emulsification technique

Two major types of emulsification were used: Direct emulsification and Phase-inversion emulsification. In these experiments, one phase was initially placed in a stirred vessel and then the second phase was gradually added to the mixing vessel. Both phases contained a constant concentration (10% *w*/*w*) of the surfactant. Depending on the phase initially placed in the vessel and also on the distribution of the surfactants between the phases six possible process approaches were identified, three for direct and three for phase-inversion emulsification. In the direct emulsification approach the dispersed phase (oil) is added to the continuous phase (water). Depending on how the surfactant mixture is incorporated into the emulsion, three combinations were examined: D1. Oil phase containing the low-HLB surfactant (Span 80) was gradually added to the water phase containing the high-HLB surfactant (Tween 80); D2. Oil phase containing half quantity of both surfactants was added to the water phase containing the rest of both surfactants; D3. Oil phase containing the high-HLB surfactant (Tween 80) was gradually added to the water phase containing the low-HLB surfactant (Span 80).

In phase-inversion emulsification, the continuous phase (water) was added to the dispersed phase (oil). Therefore, the application of such a method involves a phase inversion from an initial W/O emulsion to the desired (O/W) emulsion. Depending on how the surfactant mixture is incorporated into the emulsion, three combinations were examined: P1. Water phase containing the high-HLB surfactant (Tween 80) was gradually added to the oil phase containing the low-HLB surfactant (Span 80); P2. Water was added to the oil phase containing both surfactants; P3. The water phase containing the low-HLB surfactant (Span 80) was gradually added to the oil phase containing the high-HLB surfactant (Tween 80).

(b) Temperature

The emulsification was carried out by pre-heating separately the two phases at various temperatures, i.e., 25 ± 2 °C, 45 ± 2 °C, 70 ± 2 °C, and 85 ± 2 °C, applying the phase-inversion technique P2 and keeping the surfactants concentration at 10% *w*/*w*. 

(c) Surfactant concentration

The concentration of oil (medium chain triglycerides) and emulsion phases temperatures in all emulsions were kept constant (at 10.0 *w*/*w*% and 85 ± 2 °C, respectively), while the overall surfactant concentration (Span 80 + Tween 80) was varied from 8.0 to 12.0% *w*/*w*, keeping constant their relative ratio to achieve an HLB of 11.0. The nanoemulsion were prepared with the phase-inversion technique P2, as described above.

(d) Number of homogenization cycles

The previously characterized nanoemulsion prepared according to optimized parameters (phase-inversion emulsification P2, 10% *w*/*w* surfactant concentration and 85 °C phases temperature) were then processed with a high-pressure homogenizer, where they were subjected to 1, 3, 5, 10, and 15 homogenization cycles at a constant pressure of 1500 bar. The stability of these formulations was evaluated at predetermined storage times (up to 90 days) for macroscopic appearance, droplet size, and polydispersity index (PDI).

#### 2.2.2. Preparation of ClFPh-CHA-Loaded Nanoemulsion Preparation

In order to load the ω-3 PUFA derivative ClFPh-CHA into the optimized nanoemulsion, the emulsification process was performed as follows. Nanoemulsions were prepared by the phase-inversion method in which the water phase was continuously added into the oil phase containing both surfactants. Initially both surfactants (Span 80 and Tween 80, 3.74 and 6.26% *w*/*w* of the final preparation, respectively) were dissolved in the oil phase (10% *w*/*w*). The synthetic ω-3 PUFA derivative ClFPh-CHA was added in oil phase at the final concentration of 10 mg/mL. This ratio of surfactants was chosen to achieve an HLB value of 11.0, required HLB value necessary to obtain an oil-in-water emulsion using medium chain triglycerides as oil phase. The oil and water phases were heated separately at 85 ± 2 °C. Then, the water phase (80.0% *w*/*w*) was slowly added in the oil phase containing the blend of surfactants. The components were mixed initially using magnetic stirring at 600 rpm (model RCT, IKA^®^, Staufen, Germany) and then processed by high-pressure homogenization (Emusliflex-C5, Avestin, Ottawa, ON, Canada) until the emulsion temperature decreased to room temperature (25 ± 2 °C). 

Nanoemulsions prepared according to the determined optimal emulsification parameters were characterized for stability in terms of droplet size distribution (*z*-average and PDI) during 3 months storage.

#### 2.2.3. Nanoemulsion Characterization

(a) Macroscopic Appearance

All prepared emulsions were evaluated macroscopically by visual examination. Emulsions were carefully inspected, immediately and 24 h after preparation, in order to observe any evident macroscopic instability such as creaming or phase separation.

(b) Particle Size Distribution Analysis

Size distribution measurements were performed using a Zetasizer Nano ZS90 (Malvern Pananalytical, Malvern, UK). This system determines particle size from 2 nm up to 3.0 µm of diameter in liquid dispersions by measuring the intensity of light scattered due to the Brownian motion of the particles. Before analysis, samples were diluted 1:10 using distilled water. Dynamic Light Scattering was performed using laser wavelength 633 nm and 90° scattering angle at 25 °C. Instrument software (Zetasizer Software ver. 7.11, Malvern Pananalytical, Malvern, UK) automatically determined the most appropriate measurement duration. The hydrodynamic diameter and distribution of emulsion droplets were calculated by the cumulants fit and expressed as z-average and polydispersity index (PDI) according to International Organization for Standardization recommendations (ISO 13321:1996E “Particle Size Analysis”). All samples were evaluated in triplicate.

(c) Stability Test

Emulsions were maintained and stored at room temperature (25 ± 2 °C) for up to 90 days, monitoring their macroscopic appearance. Characterization of samples in terms of preparation particle size distribution (*z*-average and PDI) was conducted at predetermined intervals after their preparation and compared with the initial values.

(d) Determination of Encapsulation Efficiency

The encapsulation efficiency (EE%) of compound ClFPh-CHA into nanoemulsion was determined indirectly by the ultrafiltration method according to Equation (1).

(1) $$\mathrm{EE}\%=\frac{\left(\mathrm{NE}\;\mathrm{Total}-\mathrm{NE}\;\mathrm{Aqueous}\right)}{\mathrm{NE}E\;\mathrm{Total}}\times 100,$$

The percentage of encapsulated substance was calculated as the difference between the total amount of compound in the formulation, which was measured after the dissolution of the nanoemulsion in methanol (NE Total), and the amount of compound present in the aqueous phase (NE Aqueous) calculated from its concentration measured in the aqueous ultrafiltrate obtained from the nanoemulsion centrifugation (5 min, 2700× *g*, Centrifuge 5417R, Eppendorf, Macquarie Park, NSW, Australia) using centrifugal filter tubes (Ultrafree^®^ CL, cut-off 10,000 MW, Millipore, Burlington, MA, USA), divided by the total amount of compound in the nanoemulsion (NE Total) multiplied by 100. Analysis of the compound ClFPh-CHA was performed by the validated HPLC-MS method reported below.

#### 2.2.4. HPLC/MS Analytical Method for ClFPh-CHA Quantification

HPLC analysis was performed on an Agilent Technologies 6490 Triple Quadrupole LC/MS system (Agilent Technologies, Singapore) fitted with a Waters Sunfire C18 column (2.1 × 100 mm, 3.5 µm particle size, Dublin, Ireland). The samples were prepared by dissolving an aliquot of the compound in methanol. An isocratic solvent mixture of 75% acetonitrile and 25% water was employed with a flow rate of 0.5 mL/min for the first 6 min. A linear gradient to 100% acetonitrile over 2.5 min was used, held in those conditions for 1 min, and finally restored to 75% acetonitrile over 1 min and held for 1.5 min before the next injection. The total run time was 12 min. Automated sample injections (10 µL) were used, and the samples stored at 8 °C in the autosampler. Agilent Jet Stream Electrospray ionization was used in negative ionization mode with high purity nitrogen (BOC gases, Sydney, NSW, Australia) as the sheath gas at 40 psi, 2900 °C. The capillary voltage was 3500 V and the nozzle voltage was 1500 V. Selected ion monitoring was used. The data was acquired using Agilent MassHunter Workstation software (version B.06.00 SP1, Agilent Technologies, Santa Clara, CA, USA) and interpreted with the use of Microsoft Excel 2007 (Microsoft Corp., Redmond, WA, USA).

A calibration curve was constructed for ClFPh-CHA to quantify its concentration in the nanoemulsion formulation. A stock solution in methanol was prepared from an accurately weighed sample of the compound and the calibration solutions were obtained by subsequent dilution of the stock using methanol. Linearity was confirmed in the range 0.5 to 100 ng/mL (*R*^2^ = 0.9996).

#### 2.2.5. In Vivo Studies

Female Balb/c nu/nu mice (6 or 7 weeks of age) were obtained from Animal Resources Centre (Perth, WA, Australia). Mice were housed in sterile cages in a temperature-controlled animal house at the University of Sydney in accordance with the University Animal Welfare guidelines and an approved animal ethics protocol (University of Sydney Animal Ethics Committee, Approval L24/2-2012/3/5680). Mice were acclimatized and monitored in the animal house for one week prior to treatments. All mouse manipulations were carried out under aseptic conditions in a biosafety laminar flow hood. 

Human breast adenocarcinoma MDA-MB-231 cells (ATCC, Manassas, VA, USA) cultivated at 37 °C in a humidified atmosphere of 5% CO_2_ in air in DMEM supplemented with 10% fetal bovine serum (Thermo-Fisher Scientific, Scoresby, VIC, Australia) and 1% penicillin/streptomycin (Invitrogen, Carlsbad, CA, USA) were harvested using Trypsin/EDTA during exponential growth and washed twice in ice-cold PBS (pH 7.4). Cells were resuspended in ice-cold PBS combined 1:1 with Matrigel (BD Bioscience, Franklin Lakes, NJ, USA) and injected into the left inguinal mammary gland (4 × 10^4^ cells/0.1 mL). Mice were randomly assigned to either the control (CTL, *n* = 5–8) or treatment groups (*n* = 5–8). Treatment with the ω-3 17,18-epoxyeicosanoic acid isostere ClFPh-CHA by intraperitoneal (IP) or oral administration began 3–4 days after tumor cell inoculation. For intraperitoneal administration ClFPh-CHA was injected in mice at doses of 2.5, 10, or 40 mg/kg after dissolution in corn oil containing 8% DMSO (50 µL volume) once-a-day (6 days per week) for 38 days. In the case of oral administration, ClFPh-CHA-loaded nanoemulsion was administered to xenografted mice by oral gavage using 20G-38 stainless steel gavage needles at doses 2.5, 10, or 40 mg/kg (80 µL volume) once-a-day (6 days per week) and for 32 days. A control group of animals receiving vehicle only were included for each experiment.

Mouse body weights were recorded 6 days a week. Commencing eight days after tumor cell inoculation, tumor volumes were monitored by measuring the major longitudinal diameter (length, *L*) and the major transverse diameter (width, *W*) of the tumor mass using a pair of calipers every 3–4 days. The tumor volume was calculated according to Equation (2), as described previously [20].

(2) $$V=\frac{\left(L\times W^{2}\right)}{2},$$

On the final day of ClFPh-CHA administration, following euthanasia with CO_2_, all primary tumors were harvested and weighed.

#### 2.2.6. Statistics

All data are presented as mean ± standard deviation of at least three independent measures (*n* ≥ 3), if not otherwise stated. ANOVA and Fishers LSD post-hoc test was used to identify significant differences between control and ClFPh-CHA treatment groups.

## 3. Results

### 3.1. Optimization of Nanoemulsion Production Process

The initial screening of the two major emulsification techniques evidenced that only phase-inversion emulsification technique allowed for the production of stable nanoemulsions. Previous studies already evidenced that the PIT method is suitable to obtain O/A microemulsions (MEs) or nanoemulsions (NEs) with good technological properties using low percentages of non-ionic surfactants [21,22]. In this specific case, of the six compositions and phase combinations investigated, only the phase inversion emulsification process P2, in which the water phase was added to the oily phase containing both surfactants, provided a stable nanoemulsion with an average droplet size and PDI over 3 months of 130.9 ± 19.8 nm and 0.146 ± 0.02, respectively. In all other cases (D1, D2, D3, P1 and P3) phase separation occurred as soon as 24 h after preparation. 

Once evidenced that only one emulsification process provided a stable nanoemulsion, the temperature of emulsification of the two phases was investigated, as temperature affects several aspects of the emulsification process, such as solubility of surfactants, viscosity of the phases, and interfacial tension [23,24]. The particle size and stability of emulsions obtained at five different temperatures are presented in Table 1.

The nanoemulsion could not be prepared at 25 °C, as creaming of the formulation was immediately evident after 24 h. Nanometric size emulsions could be obtained for temperatures from 45 to 85 °C with sizes slightly above 100 nm and relatively narrow distribution (PDI < 0.30). Storage did not affect the properties of the nanoemulsion for up to 30 days. However, after 60 and 90 days a significant and progressive increase in droplet particle size and in polydispersity was evidenced for the nanoemulsion prepared at 70 °C, suggesting an instability due to a progressive coalescence of dispersed phase droplets. As a consequence, even though no apparent instability was evident for the preparation obtained at 45 °C, it was considered that the most robust formulation could be obtained by performing the emulsification process at 85 °C.

Subsequently, using the phase inversion emulsification procedure P2 at 85 °C, the effect of the total surfactant concentration on the characteristics of the nanoemulsions was evaluated. Table 2 presents these results in terms of nanoemulsion droplet size, PDI, and stability over 3 months.

Data evidenced that with the exception of a surfactant concentration of 8% *w*/*w*, in all other cases it was possible to obtain nanoemulsions with small particle size that were stable for at least 90 days. The formulation containing an overall surfactant concentration of 10% *w*/*w* was selected as the minimum required to provide a particle size below 150 nm and PDI below 0.2 that was stable over 90 days, suggesting a narrow droplet distribution maintained for all the three months of the stability study.

**Figure 2:**
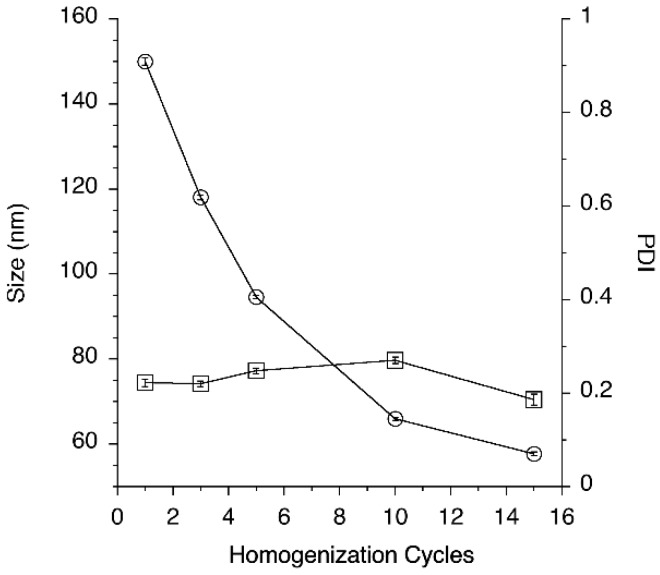
Effect of the number of homogenization cycles on nanoemulsion droplet size (empty circles) and PDI (empty squares).

Finally, the effect of the number of cycles through the high-pressure homogenizer on particle size distribution was evaluated (Figure 2).

Figure 2 shows that increasing the number of homogenization cycles progressively decreased particle size to a value slightly lower than 60 nm. At the same time PDI of nanoemulsion processed with HPH at 1500 bar was kept fairly low with values ranging between 0.186 and 0.270. The formulation processed with HPH were also found to be stable for at least 3 months at room temperature, with no relevant trend evidenced for average droplet size and PDI (data not shown). However, due to the unknown chemical stability of ClFPh-CHA to the extreme conditions of the high-pressure homogenization process, it was decided to avoid prolonged homogenization times and to limit the number of cycles to 3.

### 3.2. ClFPh-CHA-Loaded Nanoemulsion

**Figure 3:**
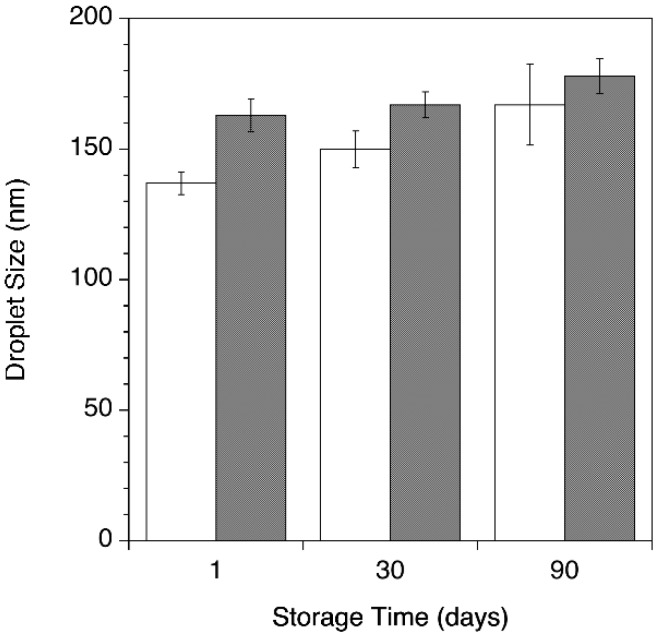
Nanoemulsion droplet size evaluated during storage for ClFPh-CHA-loaded (gray bars) and blank nanoemulsions (white bars).

As a result of the investigation of the parameters affecting the nanoemulsion preparation process, O/W nano-sized emulsions were prepared with and without ClFPh-CHA at 85 °C, with an overall concentration of surfactants of 10% *w*/*w* providing a resultant HLB of 11 and concluding the process P2 followed by 3 passages the high-pressure homogenizer. Compound ClFPh-CHA when added to the formulation, was dissolved in the oil phase to provide a final concentration of 10 mg/mL. This concentration was selected to allow the ClFPh-CHA loaded nanoemulsion to be delivered to mice in volumes of 100 µL or less. Encapsulation efficiency of 99.9 ± 2.3% suggests a complete drug encapsulation into the oil dispersed phase, as expected for a highly lipophilic compound. Nanoemulsions with and without the compound showed a milky bluish aspect with low viscosity. Nano-sized emulsions showed no macroscopic instability phenomena, such as creaming, phase separation, or drug precipitation during the storage period of 90 days at 25 °C. Average droplet size during the stability testing is shown in Figure 3.

The average droplet size of the drug-loaded nanoemulsion was greater than the blank nanoemulsion at all time points. Furthermore, droplet size did show a slight trend to increase for both drug-loaded and blank emulsion. Nevertheless, PDIs below 0.2 measured at all time during the storage time help to exclude critical phenomena of instability during storage (data not shown).

### 3.3. In Vivo Sudies in Mice Bearing an Intramammary Tumor Xenograft

**Figure 4:**
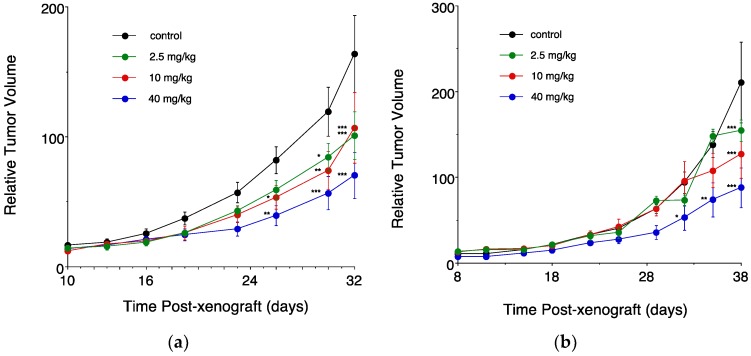
Dose-dependent effects of ClFPh-CHA on the in vivo growth of MDA-MB-231 cell xenografts. ClFPh-CHA was delivered orally as a nanoemulsion (**a**) or by intraperitoneal injection as a solution (**b**). Control groups in each experiment received vehicle only. Different from control: *** *P* < 0.001, ** *P* < 0.01, * *P* < 0.05. (Panel (**b**) reprinted with permission from [10]. Copyright (2017) American Chemical Society).

The anticancer efficacy of ClFPh-CHA was assessed in xenografted nu/nu Balb/c mice (Figure 4). As shown in Figure 4a, a significant dose-dependent decrease in tumor volume after administration of the nanoemulsion by oral gavage was evident starting from 26 days of daily treatment post-xenograft. After 32 days, significant results were obtained for all the doses tested, with the lower doses (2.5 and 10 mg/kg) reducing tumor volume to ~60% of control, and the higher dosage tested (40 mg/kg) reducing volume to 42% of control. Importantly, control and drug treated mice gained body weight throughout the study and no deaths were recorded, which indicates that ClFPh-CHA and the nanoemulsion were well tolerated.

As shown by relative tumor volume obtained for the IP administration of the drug solution (Figure 4b), a significant difference from the untreated control could be obtained only starting from day 32 and exclusively for the higher dosage. A significant tumor reduction was obtained for all the doses tested only by day 38 post-xenograft.

**Figure 5:**
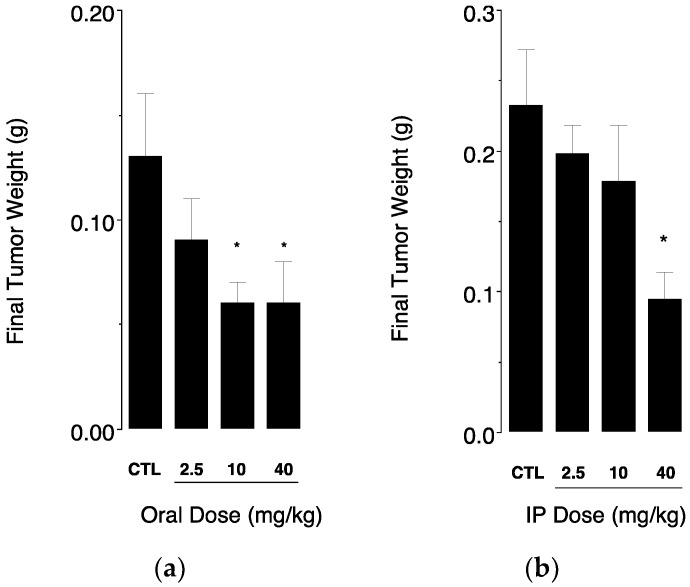
Final weights of excised tumors were determined at necropsy for (**a**) mice receiving increasing doses of ClFPh-CHA-loaded nanoemulsion orally and (**b**) mice receiving increasing doses of ClFPh-CHA in solution by intraperitoneal injection (32 days and 38 days of daily treatment for oral and IP administration, respectively). Values were compared to tumor weights of control groups of mice that received vehicle only (CTL). Different from control: * *P* < 0.05.

When considering the final weights of excised tumors (Figure 5), oral administration of the drug-loaded nanoemulsion was able to significantly reduce tumor mass to ~50% of untreated control at doses of 10 and 40 mg/kg (Figure 5a).

Interestingly, the same compound administered intraperitoneally produced a significant reduction of tumor mass to ~40% of untreated control only at the highest dose (Figure 5b), while the lower doses of 2.5 and 10 mg/kg failed to produce significant reductions in tumor mass. In addition, this result was obtained over a longer treatment period (38 days IP injection vs. 32 days oral gavage). Combined, these results suggest that oral delivery of ClFPh-CHA in the nanoemulsion is superior to IP delivery as a solution.

## 4. Discussion

Lipids and their metabolites have been identified as key mediators of cell growth and death pathways, in healthy as well as in cancer cells. Due to this role, lipid-based drug discovery in cancer research is aimed at finding new targets and develop specific and tolerable drugs able to address the proliferation, apoptosis and angiogenesis dysregulations at the base of aggressive characteristics of tumor cells [25,26,27]. We have developed a drug discovery approach based on the consideration that eicosanoids metabolites of ω-6 PUFA arachidonic acid promote tumorigenesis, while ω-3 PUFA and certain metabolites have tumor suppressive actions. Indeed, long chain ω-3 monounsaturated fatty acids were demonstrated to effectively decrease proliferation and invasiveness of breast cancer cells over-expressing COX-2, possibly via a marked decrease of ω-6 PUFA-derived eicosanoid prostaglandin E_2_ [7]. In another study, the ω-3 17,18-epoxide of eicosapentaenoic acid, but not its regioisomers, selectively showed promising anticancer properties by inducing cell cycle arrest and growth suppression through p38 MAPK activation and down-regulation of cyclin D1 [7,9]. Hence, a series of synthetic analogues of ω-3,17,18-epoxyeicosanoic acid were designed as potential drug candidates with antiproliferative and pro-apoptotic properties [8]. Among these series of analogues, ClFPh-CHA, an arylurea analogue of 17,18-epoxy-EPA, emerged as a inhibitor of breast cancer proliferation in vitro and in vivo after intraperitoneal administration [10]. Even though parenteral administration is still the main approach for cancer therapy, a major trend in recent years has been to develop formulations to allow for oral delivery of anticancer agents in order to switch to a therapy preferred by patients and providing improved quality of life. Furthermore, oral therapy is economically more convenient, avoiding the need for hospitalization, and in many studies showed less severe side effects, with patients feeling less sick and more capable to attend daily activities [28]. However, the oral delivery of many anticancer drugs presents a number of challenges because of inadequate aqueous solubility, chemical and/or enzymatic degradation in gastrointestinal fluids, extensive biotransformation in the intestinal epithelium and in the liver, and poor absorption due to poor intestinal permeability or efflux phenomena [29]. ClFPh-CHA has an extremely low aqueous solubility (~150 ng/mL), and as a consequence, is either a class II (low solubility, high permeability) or, more likely, a class IV (low solubility, low permeability) substance according to the Biopharmaceutical Classification System (BCS); this predicts dissolution rate-limited intestinal absorption and variable absorption from the GI tract [30,31]. ClFPh-CHA required a formulation that enabled oral delivery and to assure its bioavailability via this administration route. Among possible options, nano-sized drug delivery systems encapsulating lipophilic drugs have been demonstrated to be able to protect the drug from GI degradation, enhance drug absorption through the intestinal barriers, and ultimately enhance bioavailability by modifying the pharmacokinetic profile of the encapsulated drug [32]. In particular lipid-based nanomedicines such as liposomes [33], solid lipid nanoparticles [34], and polymer/lipid hybrid systems [35,36] have shown potential through a number of mechanisms ranging from mucoadhesion and apparent drug dissolution in GI tract fluids to the modification of enterocyte-based transport and metabolism and even providing selective lymphatic uptake of the drug [37].

In the present work, a straightforward approach was selected with the development of a nanoemulsion able to encapsulate ClFPh-CHA and presenting particle size below 200 nm and narrow particle size distribution. The formation of nano-sized o/w emulsions was attributed to the kinetics of emulsification during the catastrophic phase-inversion process and the consequent rearrangement of the interfacial surfactant molecules resulting in curvature change and fine dispersion of the dispersed phase. It was decided to use two different surfactants (Tween 80 and Span 80) because it has been suggested that a single surfactant is unlikely to produce the desired stability [23]. The combination of the composition of the initial phases, temperature and surfactant concentration produced a stable nanoemulsion suitable for loading with a relatively high drug concentration. High pressure homogenization was selected as a convenient scale-up method to further refine the nanoparticle droplet size distribution, since this could have an impact on GI tract lipolysis and the enhancement of bioavailability [38].

More significantly, when administered orally every day to mice bearing breast cancer xenografts, the nanoemulsion showed a significant reduction in tumor volume after 26 days for the two higher doses tested (10 and 40 mg/kg) and after 30 days also for the lowest (2.5 mg/kg) when compared to tumors developed in untreated mice. This preliminary in vivo finding demonstrated that the drug, already shown to be an effective pro-apoptotic compound after intraperitoneal injection, is absorbed when administered orally as a nanoemulsion and presumably achieves blood concentrations sufficient to decrease MDA-MB-231 cells proliferation. Furthermore, these cells are human triple-negative breast cancers cells, i.e., not expressing the molecular targets used in the treatment of other breast cancers. Triple-negative cancers are more difficult to treat and their prognosis is extremely poor [1]. Further studies should determine the absolute bioavailability and the mechanism of absorption of ClFPh-CHA. However, considering the highly lipophilic nature of the compound it is possible that its inclusion in the nanoemulsion favored its lymphatic uptake [39]. In fact, other lipid-based nanocarriers such as liposomes and solid lipid nanoparticles have been shown to be suitable carriers for lymphatic delivery [40]. The drug, absorbed through this physiologic intestinal lipid transport system, would reach the systemic circulation through lymphatic vessels and draining lymph nodes, bypassing the liver and first-pass metabolism, and ultimately enhancing its oral bioavailability.

Interestingly, when the tumor weight was taken into account, ClFPh-CHA-loaded nanoemulsion administered orally produced a significant reduction of tumor mass at a dose of 10 mg/kg, while a dose of 40 mg/kg was required for a similar reduction in tumor mass when administered intraperitoneally. This finding suggests that the efficacy of the drug could be greater after oral administration. One explanation could be that the bioavailability is suboptimal after intraperitoneal injection since the injection of the corn oil/DMSO solution could lead to drug precipitation when in contact with peritoneal fluids. In fact, an abdominal precipitate was evident in ClFPh-CHA-treated rats at necropsy after administration of the 40 mg/kg dose. Drug precipitation would lead to delayed absorption due to slow drug crystals dissolution and low drug concentrations at a tumor site.

## 5. Conclusions

Nanotechnologies have been often applied the administration of existing anticancer drugs, with the aim to modify their pharmacokinetics, improve their efficacy, and reduce drug-related adverse effects. This reformulation strategy often improves the clinical utility of old drugs offering important benefits such as reduction of critical toxicities [41]. In this work, pharmaceutical nanotechnologies were applied to the formulation of a promising lipid-based anticancer drugs designed to provide a valid alternative to existing treatment to aggressive forms of breast cancer. A nanoemulsion formulation approach was selected to enable the innovative anticancer drug oral delivery, despite its limited aqueous solubility. In vivo preliminary results indicate that ω-3 17,18-epoxyeicosanoic acid bioisosteres can be formulated as oil-in-water nanoemulsion providing sufficient absorption and bioavailability to hinder tumor proliferation to an extent matching, if not exceeding, the anticancer activity obtained via a conventional parenteral administration.

## Figures and Tables

**Table 1:** Influence of emulsification temperature on droplets size distribution and stability.

Time	1 Day		30 Days		60 Days		90 Days	
*T* (°C)	Size (nm)	PDI	Size (nm)	PDI	Size (nm)	PDI	Size (nm)	PDI
25	Creaming		-	-	-	-	-	-
45	101.5 ± 1.1	0.17 ± 0.02	125.9 ± 2.4	0.20 ± 0.00	113.8 ± 1.2	0.23 ± 0.01	104.5 ± 0.4	0.17 ± 0.01
70	133.1 ± 8.0	0.29 ± 0.04	135.4 ± 8.3	0.29 ± 0.01	243.4 ± 29.7	0.28 ± 0.08	554.5 ± 11.2	0.44 ± 0.01
85	137.1 ± 3.0	0.16 ± 0.01	144.6 ± 3.1	0.16 ± 0.01	136.9 ± 0.8	0.13 ± 0.02	150.5 ± 0.3	0.24 ± 0.01

**Table 2:** Influence of the total concentration of surfactants on droplet size distribution and stability.

Time	1 Day		30 Days		60 Days		90 Days	
Surf. (%) ^1^	Size (nm)	PDI	Size (nm)	PDI	Size (nm)	PDI	Size (nm)	PDI
8	162.4 ± 7.7	0.28 ± 0.02	Coalescence		-	-	-	-
9	150.0 ± 1.1	0.20 ± 0.02	156.5 ± 3.2	0.15 ± 0.02	164.4 ± 3.2	0.25 ± 0.04	151.1 ± 1.8	0.19 ± 0.01
10	137.1 ± 3.0	0.16 ± 0.01	144.6 ± 3.1	0.16 ± 0.01	136.9 ± 0.8	0.13 ± 0.02	150.5 ± 0.3	0.24 ± 0.01
11	120.3 ± 2.3	0.10 ± 0.03	130.7 ± 6.2	0.14 ± 0.02	125.0 ± 1.3	0.13 ± 0.02	122.5 ± 1.1	0.17 ± 0.01
12	122.3 ± 1.5	0.10 ± 0.02	129.0 ± 3.3	0.12 ± 0.02	126.4 ± 1.1	0.18 ± 0.01	124.9 ± 0.5	0.17 ± 0.01

^1^ Overall percentage by weight of Span 80 and Tween 80.
